# Coordinated miRNA/mRNA Expression Profiles for Understanding Breed-Specific Metabolic Characters of Liver between Erhualian and Large White Pigs

**DOI:** 10.1371/journal.pone.0038716

**Published:** 2012-06-12

**Authors:** Runsheng Li, Qinwei Sun, Yimin Jia, Rihua Cong, Yingdong Ni, Xiaojing Yang, Zhihua Jiang, Ruqian Zhao

**Affiliations:** 1 Key Laboratory of Animal Physiology & Biochemistry, Ministry of Agriculture, Nanjing Agricultural University, Nanjing, Jiangsu, China; 2 Department of Animal Sciences, Washington State University, Pullman, Washington, United States of America; University of Queensland, Australia

## Abstract

MicroRNAs (miRNAs) are involved in the regulation of various metabolic processes in the liver, yet little is known on the breed-specific expression profiles of miRNAs in coordination with those of mRNAs. Here we used two breeds of male newborn piglets with distinct metabolic characteristics, Large White (LW) and Erhualian (EHL), to delineate the hepatic expression profiles of mRNA with microarray and miRNAs with both deep sequencing and microarray, and to analyze the functional relevance of integrated miRNA and mRNA expression in relation to the physiological and biochemical parameters. EHL had significantly lower body weight and liver weight at birth, but showed elevated serum levels of total cholesterol (TCH), high-density lipoprotein cholesterol (HDLC) and low-density lipoprotein cholesterol (LDLC), as well as higher liver content of cholesterol. Higher serum cortisol and lower serum insulin and leptin were also observed in EHL piglets. Compared to LW, 30 up-regulated and 18 down-regulated miRNAs were identified in the liver of EHL, together with 298 up-regulated and 510 down-regulated mRNAs (FDR<10%). RT-PCR validation of some differentially expressed miRNAs (DEMs) further confirmed the high-throughput data analysis. Using a target prediction algorithm, we found significant correlation between the up-regulated miRNAs and down-regulated mRNAs. Moreover, differentially expressed genes (DEGs), which are involved in proteolysis, were predicted to be mediated by DEMs. These findings provide new information on the miRNA and mRNA profiles in porcine liver, which would shed light on the molecular mechanisms underlying the breed-specific traits in the pig, and may serve as a basis for further investigation into the biological functions of miRNAs in porcine liver.

## Introduction

MicroRNAs (miRNAs) are short non-coding RNAs (about 22 nucleotides) which play important roles in post-transcriptional regulation by mRNA cleavage and/or translational repression [1]. miRNAs are involved in almost every biological process, including cell growth and differentiation [2], pathogenesis and disease prevention [3]. In mammals, temporal and spatial changes in miRNA expression have been well characterized [4] to delineate the miRNA transcriptomes of different tissues [2], [5], [6] at different development stages [7]. Also, miRNA expression profiles were elaborated on the same tissue derived from different animal breeds or species. For example, the muscle miRNA expression profiles were compared between broiler and layer chickens to understand the role of miRNAs in myogenesis [8], and miRNAs expressed in the skin of goat and sheep were profiled to study the role of miRNAs in wool growth [9].

Pig is one of the most important domestic species for meat production [10] and can also serve as an ideal model for biomedical studies on various metabolic disorders, such as obesity [11] and cardiovascular diseases [12], [13] in humans. Great efforts have been made to sequence and decode the genome [14] and to identify the miRNAs [15], [16] in the pig. As of November 2011, the microRNA database, miRbase (Release 18.0, http://www.mirbase.org) contains 257 mature pig miRNAs. The majority of these miRNAs are conserved among mammals, only few are pig specific. Recently, Solexa deep sequencing technology was employed not only to reveal the porcine miRNA transcriptome (microRNAome) in multiple tissues [6], [7], but also to investigate the ontogeny of miRNA expression in the pig at different developmental stages [16], [17].

Erhualian (EHL) is a Chinese indigenous pig breed, being known for its early sexual maturity, large litter size, high adiposity, mild temper, good maternity and high tolerance to roughage and stress [18]. In our previous study, we found distinct behavioral, endocrine and biochemical response patterns during transportation between Chinese indigenous breed and Western breed [19]. So far, mRNA transcriptomes in placenta and ovary of Chinese indigenous pigs have been profiled to understand the molecular mechanisms involved in their high prolificacy [20], [21]. However, the study of hepatic mRNA transcriptome in EHL is still lacking, and the link between mRNA transcriptome and the metabolic parameters in the pig has not been well established.

Liver is a key metabolic organ and thus often being chosen as a target for miRNA profiling to understand metabolic regulations. For example, obese diabetic model mice such as ob/ob, db/db and KKAy were reported to express higher miR-335 in the liver compared to normal mice [22]. Inhibition of miR-122 in liver resulted in reduced plasma cholesterol levels, accompanied by a decrease in hepatic fatty-acid and cholesterol synthesis rate [23]. For genome-wide analysis, integrating differentially expressed miRNAs (DEMs) with differentially expressed genes (DEGs) should present a comprehensive way to study their functions in metabolomes. Some integrated analyses of liver miRNA and mRNA have been carried out in model animals [24]. However, coordinated analysis of hepatic miRNA and mRNA expression profiles in relation with the metabolic characteristics of different breeds of pigs is lacking.

Here we used male newborn piglets of Large White (LW) and EHL to delineate the expression profiles of mRNA with microarray and miRNA with both deep sequencing and microarray, and to analyze the functional relevance of integrated miRNA and mRNA expression in relation with the physiological and biochemical characters. Solexa deep sequencing was employed to get a full scope of porcine liver miRNAome and to identify the differentially expressed miRNAs between the two pig breeds. A miRNA microarray containing 238 probes and RT-qPCR were used to supplement and confirm DEMs. The mRNA expression profile was evaluated by microarray analysis. The miRNAome and transcriptome were integrated and the functional relevance was analyzed linking breed-specific metabolic phenotypes in EHL and LW pigs.

## Results

### Metabolic Characteristics of Two Pig Breeds

As shown in Table 1, body weight, body length and liver weight of newborn EHL piglets were significantly lower than those of LW (FDR <0.05), yet the liver index remained unchanged (FDR >0.05). Serum total cholesterol (TCH), high density lipoprotein cholesterol (HDLC) and low density lipoprotein cholesterol (LDLC) in EHL piglets were significantly higher than those in LW piglets (FDR <0.05), while serum glucose and triglyceride (TG) did not differ between the two pig breeds. The TCH content in liver was also higher in EHL, while liver content of TG showed no difference between breeds. Serum cortisol concentration was significantly increased (FDR <0.05), while serum leptin and insulin levels were significantly decreased (FDR <0.05) in EHL piglets compared to those in LW. Serum triiodothyronine (T3), thyroxine (T4), free T3 (FT3) and T3/T4 ratio showed no difference between the two breeds (Table 1).

**Table 1:** Metabolic and endocrine parameters in two breeds of piglets.

Parameters	LW	EHL	p-value	FDR
Body length (cm)	25.83±0.31	19.67±0.92	0.001	0.004
Body weight (kg)	1.34±0.03	0.76±0.03	0.000	0.000
Liver weight (g)	32.87±1.57	16.19±0.85	0.000	0.000
Liver index (g/kg)	24.50±1.04	21.56±1.60	0.153	0.251
Serum leptin (ng/ml)	5.06±0.33	3.61±0.35	0.015	0.038
Serum insulin (µIU/ml)	18.91±2.61	11.12±1.37	0.025	0.044
Serum cortisol (ng/ml)	152.84±26.65	297.08±45.63	0.019	0.038
Serum FT3 (fmol/ml)	4.29±1.06	3.36±0.34	0.391	0.470
Serum T3 (ng/ml)	4.44±0.37	4.87±0.19	0.318	0.441
Serum T4 (ng/ml)	38.15±8.97	37.6±2.47	0.953	0.953
T3/T4	0.18±0.06	0.13±0.01	0.477	0.551
Serum Glucose (mmol/L)	3.17±0.29	2.79±0.32	0.391	0.470
Serum TG (mmol/L)	0.29±0.07	0.27±0.06	0.796	0.842
Serum TCH (mmol/L)	0.77±0.03	1.55±0.16	0.004	0.016
Serum HDLC (mmol/L)	0.31±0.02	0.44±0.04	0.017	0.038
Serum LDLC (mmol/L)	0.24±0.02	0.55±0.08	0.014	0.019
Liver TCH (mg/g)	1.61±0.33	2.69±0.13	0.012	0.036
Liver TG (mg/g)	5.33±1.18	3.90±0.51	0.300	0.441

All data were expressed as mean ± SEM, and FDR <0.05 was considered significant.

### Liver miRNA Expression Profiling of Two Pig Breeds by Deep Sequencing, Microarray and RT-PCR Validation

After trimming of adaptor sequences and removal of reads containing ambiguous base calls, the sequence reads were clustered into unique sequences. In total, there were 25,957,969 and 26,574,154 trimmed reads in two libraries, with 15,154,209 (58.3% of trimmed reads) and 19,099,533 (71.8% of trimmed reads) mappable reads that aligned to unique miRNAs for the pooled liver samples of LW and EHL in the range of 19–25 nt. The read size mainly ranged from 21 to 23 nt. The percentage of the 22 nt reads in total reads was 64.2% for LW and 64.0% for EHL. The top 10 highest expressed miRNAs detected by deep sequencing were miR-148a, miR-101, miR-143-3p, miR-122, miR-30a-5p, miR-21, miR-30c, miR-192, miR-27b and miR-24 (Table S1).

**Table 2:** Differentially expressed miRNAs in the liver between Large White and Erhualian piglets detected with deep sequencing compared to microarray and RT-PCR validation.

	microRNA sequencing			microarray			RT-qPCR	
miRNA	RPM (LW)	RPM (EHL)	log2 Ratio (EHL/LW)	log2 Ratio (EHL/LW)	p-value	FDR(%)	log2 Ratio (EHL/LW)	p-value
*down-regulated miRNAs*								
miR-146a	31	19	−0.71	#	#	#	−0.30	0.380
miR-21*	22	15	−0.63	–	–	–		
miR-222	1050	688	−0.61	−0.35	0.566	35.993	−0.99	0.008
miR-2887	49	23	−1.08	–	–	–		
miR-2904	10	6	−0.76	–	–	–		
miR-2904-3p	11	7	−0.62	–	–	–		
miR-375	2315	1549	−0.58	–	–	–		
miR-4332-3p	319	139	−1.20	0.24	0.513	62.118		
miR-4332-5p	25	9	−1.52	–	–	–		
miR-485-3p	34	18	−0.93	–	–	–		
miR-574	1072	570	−0.91	−0.77	0.252	18.188	−0.69	0.121
miR-652	33	16	−1.06	#	#	#	−1.28	0.010
miR-739	17	10	−0.77	–	–	–		
miR-874	297	189	−0.65	–	–	–		
miR-874*	19	12	−0.65	–	–	–		
*up-regulated miRNAs*								
miR-100	746	1170	0.65	0.13	0.737	62.17	0.42	0.379
miR-129	12	22	0.87	#	#	#		
miR-133a	67	129	0.95	#	#	#	1.58	0.020
miR-1343	14	21	0.60	–	–	–		
miR-140	427	666	0.64	0.8	0.19	34.194		
miR-144	41	86	1.07	–	–	–		
miR-155	133	585	2.14	#	#	#		
miR-184	21	59	1.48	1.71	0.009	0		
miR-188-5p	69	141	1.03	–	–	–		
miR-190	69	124	0.84	–	–	–		
miR-193a-3p	13	32	1.24	#	#	#		
miR-193a-5p	13	24	0.83	1.13	0.032	6.839		
miR-193b*	6	10	0.64	–	–	–		
miR-19a	64	97	0.59	#	#	#		
miR-210	16	31	0.96	#	#	#		
miR-216	3	19	2.71	#	#	#	1.31	0.000
miR-335	41	67	0.72	#	#	#		
miR-335*	32	49	0.62	–	–	–		
miR-362	83	143	0.79	#	#	#		
miR-451	2056	3165	0.62	0.12	0.713	40.884		
miR-582-3p	5	13	1.48	–	–	–		
miR-582-5p	12	33	1.42	–	–	–	1.01	0.060
miR-590	65	106	0.70	–	–	–		
miR-660	335	509	0.60	–	–	–		
miR-802	12	21	0.81	–	–	–		

The miRNA expression in the liver of Erhualian piglets was compared with that of Large White. miRNAs with fold change >1.5 and average RPM >10 are shown. “#" means that this miRNA could not be detected by microarray due to low fluorescence, while “–" means that the probe for the miRNA is missing on the microarray.

Counts in reads per million (RPM) was used to quantify miRNA expression in the liver of EHL and LW piglets. These unique sequences with RPM >10 were annotated based on their similarities with mature miRNA sequences published in miRBase (release 18.0), resulting in a list of 211 mature miRNAs (Table S1). miRNAs with less than 2 nucleotide substitution outside seed region were considered as one miRNA family [25], [26], and the copy numbers of all miRNAs in this family were added together for reporting read counts and for differential expression calculations. miRNAs with fold change >1.5 and at least 10 RPM average expression (in both pig breeds) were selected as differentially expressed miRNAs (DEMs).

**Table 3:** Differentially expressed miRNAs in the liver between Large White and Erhualian piglets detected with microarray compared to the deep sequencing and RT-qPCR validation.

	microarray			microRNA Sequencing				RT-qPCR	
miRNAs	log2 Ratio(EHL/LW)	p-value	FDR(%)	RPM(LW)		RPM(EHL)	log2 Ratio(EHL/LW)	log2 Ratio(EHL/LW)	p-value
*down-regulated miRNAs*									
miR-15b	−0.44	0.033	12.823	<10	<10			−0.79	0.022
miR-221	−0.80	0.069	12.823	2202	1781		−0.31	0.29	0.418
miR-27a	−0.83	0.048	12.823	331	389		0.23	−0.22	0.314
*up-regulated miRNAs*									
miR-130b	0.72	0.079	6.839	175	220		0.33	0.87	0.002
miR-184	1.71	0.009	0	21	59		1.48	0.61	0.001
miR-185	0.78	0.035	6.839	<10	<10			0.79	0.027
miR-193a-5p	1.13	0.032	6.839	13	24		0.88	1.33	0.038
miR-378	1.07	0.09	6.839	903	1249		0.47	1.12	0.001
miR-500	0.79	0.091	12.823	907	1299		0.52	0.47	0.080
miR-532-5p	1.37	0.086	6.839	1600	2202		0.46	1.93	0.025

The miRNA expression in the liver of Erhualian piglets was compared with that of Large White. miRNAs with FDR <15% are shown. “<10" means the miRNA with RPM less than 10 is not shown in the table.

Compared to LW, EHL demonstrated 40 DEMs (15 down-regulated and 25 up-regulated) by sequencing (Table 2) and 10 DEMs (3 down-regulated and 7 up-regulated) by microarray (Table 3). Among the 40 DEMs obtained by deep sequencing, 8 (miR-100, miR-140, miR-184, miR-193a-5p, miR-222, miR-4332-3p, miR-451, and miR-574) were detectable in microarray. The fold changes of these miRNAs, except miR-4332-3p, were consistent between the two methods, but only miR-184 and miR-193a-5p were also identified as DEMs by microarray. The remaining 32 DEMs identified by deep sequencing were not confirmed by the microarray analysis. Among them, 21 DEMs were missing their specific probes on the microarray which was designed according to an earlier version of pig miRbase (release 16.0), while the other 11 DEMs were undetectable due to low hybridization signals. Eight DEMs, 4 down-regulated (miR-146a, miR-222, miR-574 and miR-652) and 4 up-regulated (miR-100, miR-133a, miR-216 and miR-582-5p), were randomly chosen for validation using RT-qPCR. Among the 8 DEMs detected by deep sequencing, 4 were proved significant (p<0.05) by qPCR, one had a tendency to be significant (p = 0.06), yet 3 (miR-146a, miR-574 and miR-100) were not confirmed significant (Table 2).

All the 7 up-regulated miRNAs identified by microarray had an EHL/LW ratio >1.2 (log2 (EHL/LW) >0.26) in deep sequencing, and were all confirmed by RT-qPCR. However, among the 3 down-regulated miRNAs, only miR-221 was consistent with the sequencing data, while only miR-15b was confirmed with RT-qPCR. All together, we identified 30 up-regulated and 18 down-regulated miRNAs in the liver of EHL piglets with sequencing and microarray.

### Liver mRNA Expression Profiling of Two Pig Breeds by Microarray

Six pig transcriptome microarrays were performed for hepatic gene expression profiling in EHL and LW piglets. Among 44,987 pig transcripts investigated, 23,807 transcripts were retained for further analysis after removing the probes with poor quality intensities and low dependability. All these qualified transcripts were then annotated by manual blast referring to previously developed protein-based annotation for pigs [27], [28]. In total, 9,447 genes were found to be expressed in the liver of newborn piglets.

**Figure 1:**
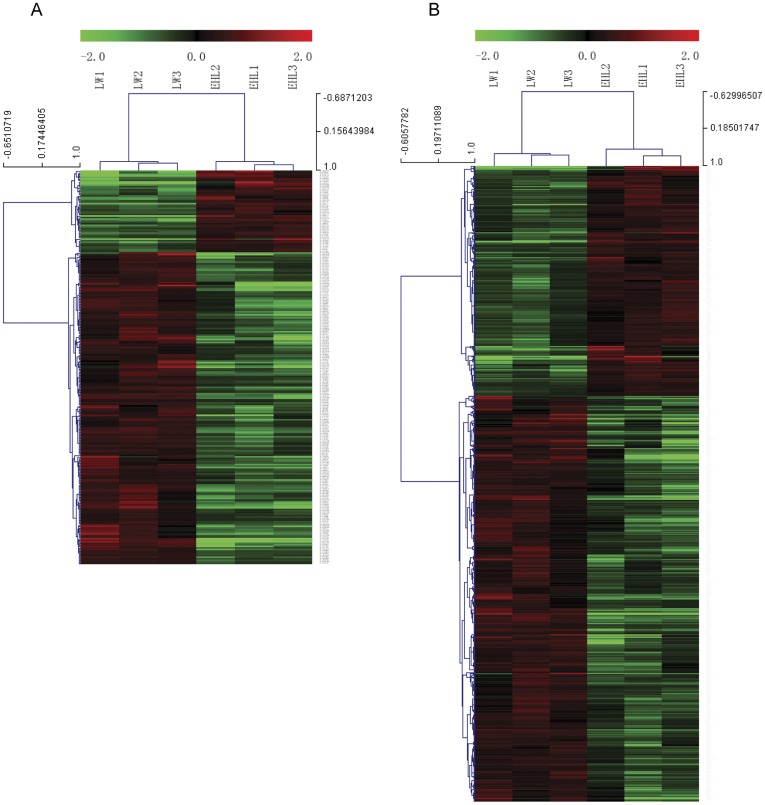
Hierarchical cluster analysis of the differentially expressed mRNAs in liver from Large White (LW) and Erhualian (EHL) piglets. The figure was drawn by MeV software (version 4.2.6). (A) Differentially expressed mRNAs chosen with FDR <5%; (B) Differentially expressed mRNAs chosen with FDR <10%. Correlation (uncentred) similarity matrix and average linkage algorithms were used in the cluster analysis. Each row represents an individual mRNA, and each column represents a sample. The dendrogram at the left side and the top displays similarity of expression among mRNAs and samples individually. The color legend at the top represents the level of mRNA expression, with red indicating high expression levels and green indicating low expression levels. The codes on the legend are log2-transformed values.

The microarray data of LW piglets were treated as control in the selection of differentially expressed genes related to EHL piglets. After the removal of redundant and unannotated sequences, with FDR <5%, 53 genes were found to be significantly up-regulated and 200 genes to be significantly down-regulated in the EHL piglets compared to the LW piglets (Fig. 1A). With FDR <10%, 298 genes were found to be significantly up-regulated and 510 genes to be significantly down-regulated in the EHL piglets (Fig. 1B).

### Coordinated Expression Patterns between miRNAs and mRNAs

As described above, the DEGs were selected based on either FDR <5% or FDR <10% (Figure 1), while the DEMs were chosen based on either sequencing (fold change >1.5) or microarray (FDR <15%) data.

**Figure 2:**
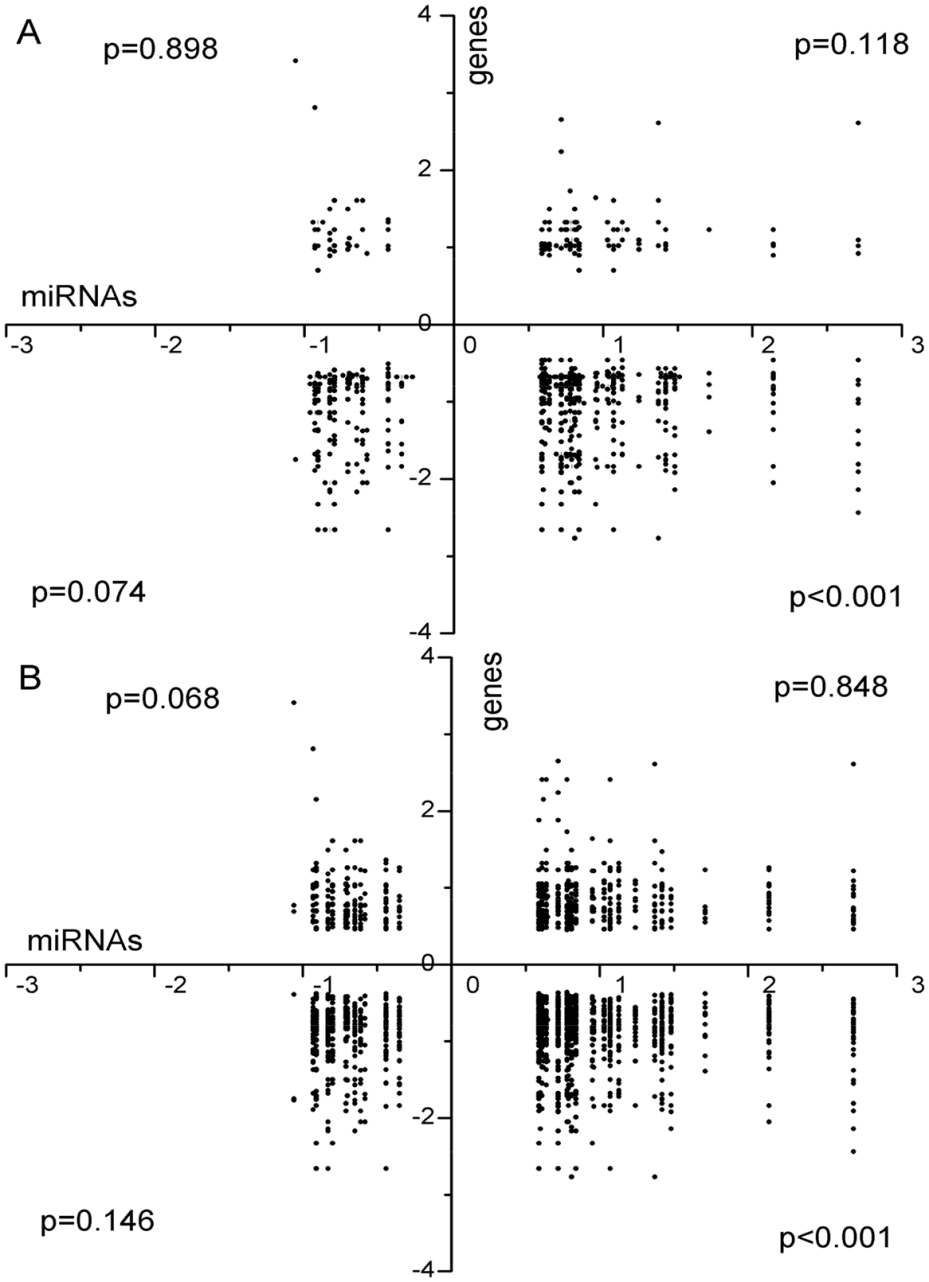
Targets of DEMs among DEGs. (A) Targets of DEMs included in DEGs list with FDR <5%; (B) Targets of DEMs included in the DEGs list with FDR <10%. Targets of DEMs were predicted by miRanda method. The target genes which were included in the DEGs were plotted. The x axis is log2 transformed fold change of DEMs, while the y axis stands for log2 transformed fold change of DEGs. The p-value was assessed by a two tailed chi-square test.

In this study, the target gene lists of miRNAs predicted by miRanda (http://www.microrna.org/microrna/) based on the human sequences were used. Unfortunately, 10 miRNAs including 4 up-regulated (miR-129*, miR-1343, miR-193b* and miR-590) and 6 down-regulated (miR-2887, miR-2904, miR-2904-3p, miR-4332-3p, miR-4332-5p and miR-739) do not have orthologous genes in human, so they do not have target genes in the database. As such, these miRNAs were excluded for further analysis. The remaining 12 down-regulated and 26 up-regulated miRNAs were associated with 170 and 417 gene targets, respectively, in the DEG list selected at FDR <5% (Figure 2A). Among 170 genes targeted by 12 down-regulated miRNAs, 32 (19%) were up-regulated and 138 (81%) were down-regulated. Among 417 genes targeted by 26 up-regulated miRNAs, 336 (81%) were down-regulated, and 81 (19%) were up-regulated. Similar patterns of miRNA-mRNA association were seen with the DEG list selected at FDR <10% (Figure 2B). Among 478 genes targeted by down-regulated miRNAs, 151 (32%) were up-regulated and 327 (68%) were down-regulated. Among 1,277 genes targeted by up-regulated miRNAs, 377 (30%) were up-regulated and 900 (70%) were down-regulated. A two tailed chi-square test was conducted to determine whether the number of predicted targets of DEMs was significantly higher than that would be expected by chance. The significance of all the 4 types of possible miRNA-mRNA correlations was analyzed. It turned out that only the correlation of up-regulated miRNAs and down-regulated genes was statistically significant (p<0.01).

**Figure 3:**
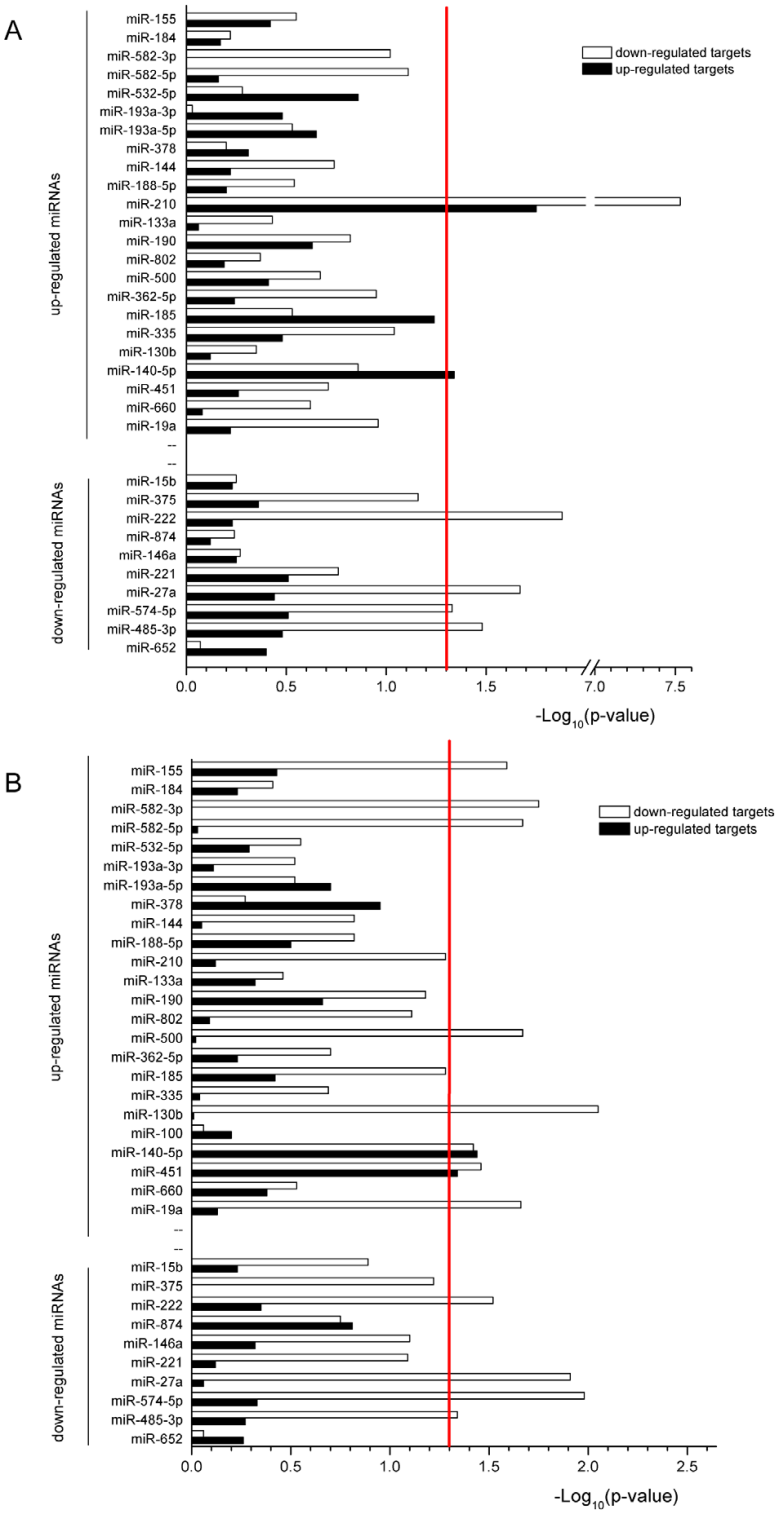
The targets of each individual DEM included in the lists of DEGs. (A) DEGs with FDR <5%; (B) DEGs with FDR <10%. A two-tailed Fisher’s Exact Test was used to determine the significance (p<0.05, above red line at 1.3). The negative log of the p-value is plotted on the x-axis for both down-regulated mRNAs (white) and up-regulated genes (black).

To identify whether a miRNA was negatively correlated with predicted target DEGs, the number of down- and up-regulated targets of a DEM was compared with the number of stay-still targets (with FDR >10%) by two-tailed Fisher’s Exact Test. The results of this analysis were presented in Figure 3. When using the DEG list of FDR <5%, only miR-210 had a significant reciprocal expression with its targets. When using the DEG list of FDR <10%, 10 of 26 up-regulated miRNA had a significant higher number of target mRNAs that were down-regulated. The differences obtained by using different DEG lists imply that the target of a miRNA may usually be fine-tuned. When testing miRNAs that were down-regulated, no miRNAs had a significant number of target genes that were up-regulated, but 4 miRNAs (miR-222, miR-27a, miR-574-5p, and miR-485-3p) had a significant number of target genes that were also down-regulated when using both DEG lists of <5% and <10%.

### Functional Analysis of DEGs

**Figure 4:**
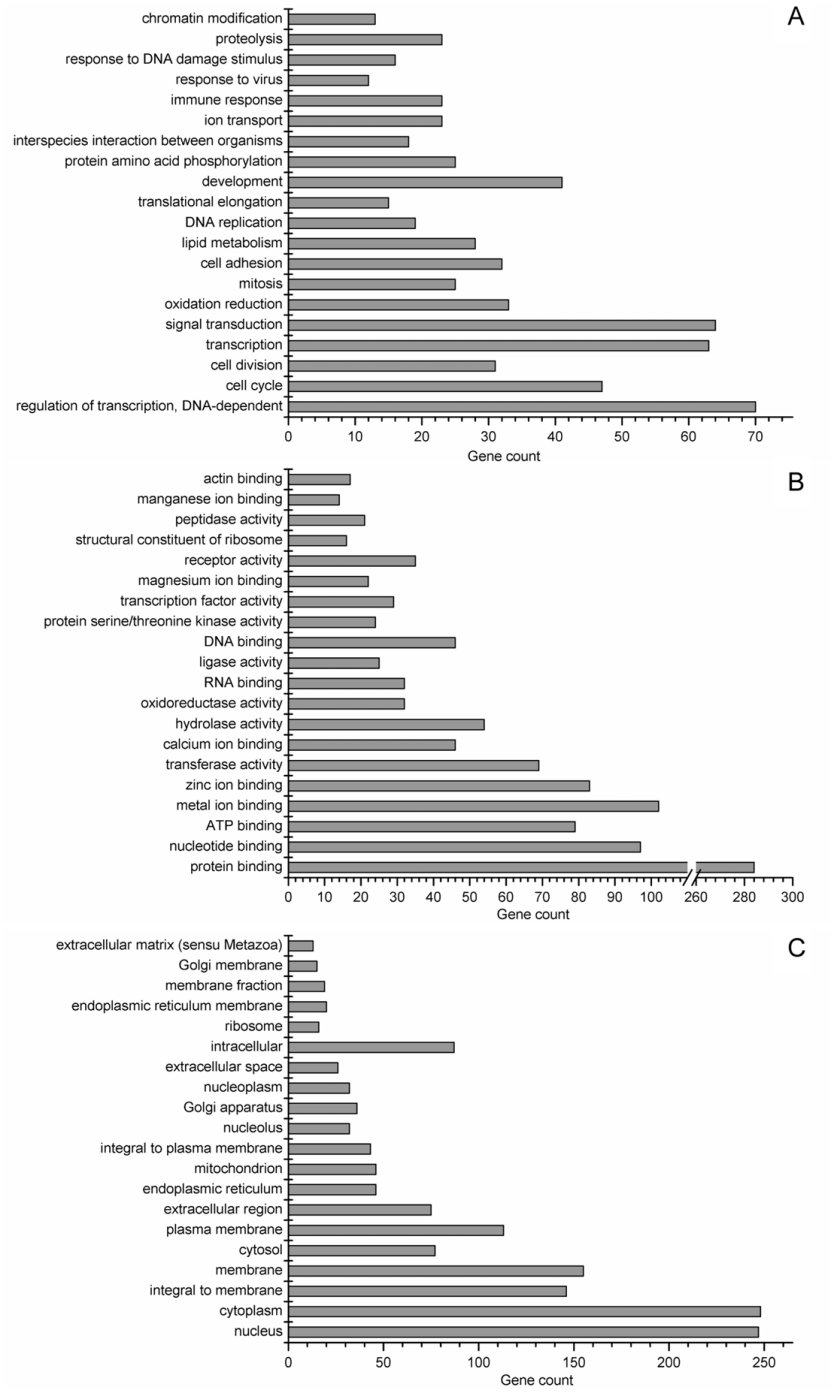
Gene ontology analysis of DEGs with FDR <10%. (A) biological process; (B) molecular function; and (C) cellular component. The GO terms were sorted by the enrichment p-value calculated by MAS 3.0, in an ascending order from bottom to top.

To define the biological functions of all the 808 DEGs (Table S2) selected at FDR <10%, the gene ontology (GO) analysis and KEGG pathway analysis (Table S3) were carried out. GO terms were sorted by p-value, in an ascending order from bottom to top (Figure 4). For the biological process, liver functions between LW and EHL mainly differ in regulation of transcription, cell cycle (the GO term of cell cycle, cell division and mitosis), transcription, signal transduction, oxidation reduction, cell adhesion, lipid metabolism, DNA replication, translational elongation, development, protein amino acid phosphorylation, interspecies interaction between organisms, ion transport, immune response, response to virus, response to DNA damage stimulus, proteolysis and chromatin modification.

**Figure 5:**
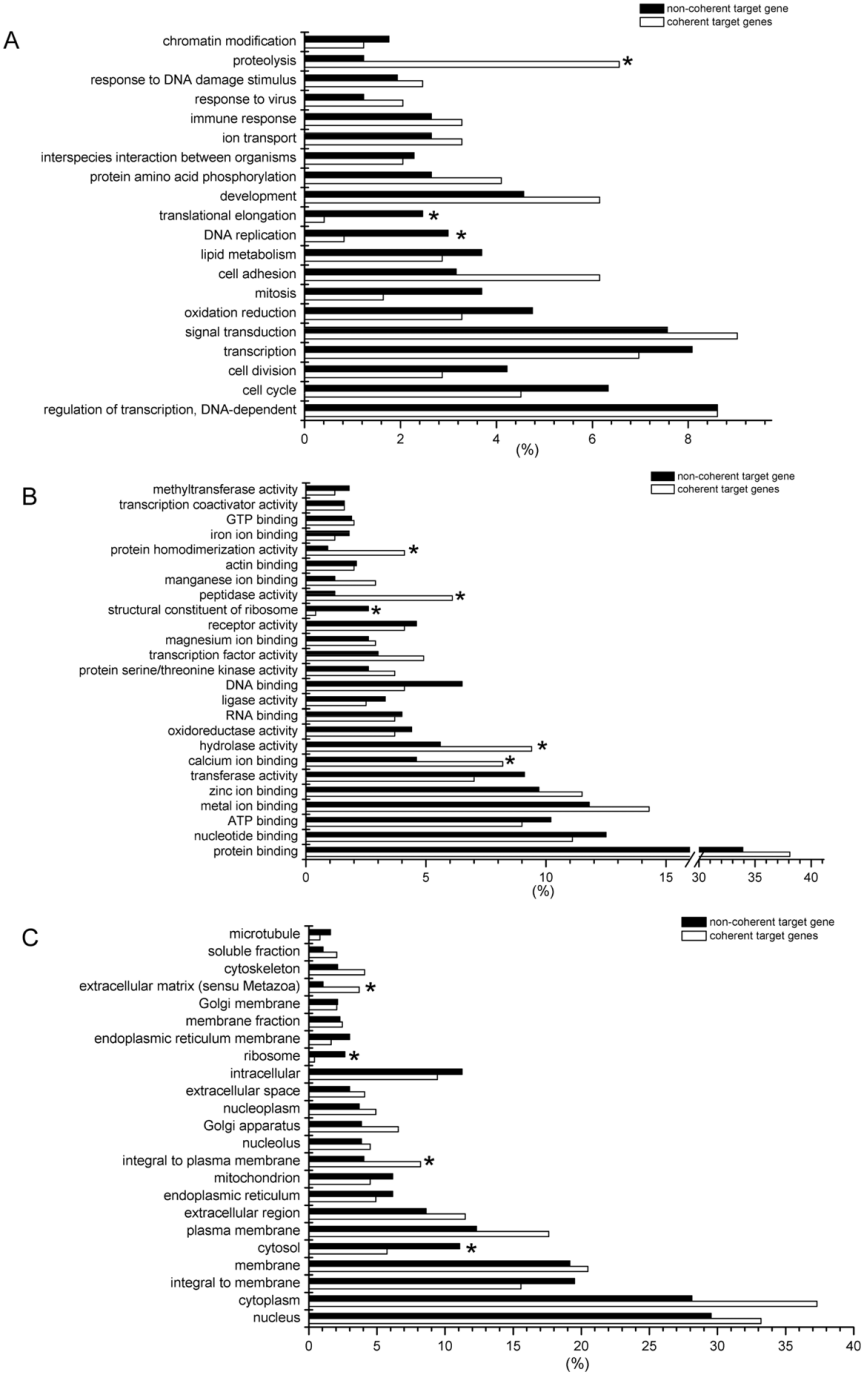
Gene ontology analysis of miRNA targeted DEGs and non-targeted DEGs. (A) biological process; (B) molecular function; and (C) cellular component. The x axis represents the percentages of genes in total targeted or non-targeted DEGs of each GO term. The p-values assigned to GO terms were calculated by chi-square test, * indicates p-value <0.05.

To characterize the function of miRNA-mediated DEGs, we used the target DEGs which are reversely expressed with DEM for GO analysis. Using the DEGs with FDR <10%, we observed 229 coherent targets and 579 non-coherent target genes. The percentages of coherent target and non-coherent target genes involved in each GO term were and compared (Figure 5). When considering biological process, the genes involved in proteolysis were significantly enriched among the target genes. The enrichment of proteolysis is concordant with the enrichment of hydrolase activity, peptidase activity and protein homodimerization activity in molecular function analysis. Meanwhile, DEGs involved in DNA replication, translational elongation and the constituent of ribosome are barely miRNA-mediated.

## Discussion

In this study we observed significant differences in physiological and biochemical traits between newborn male EHL and LW piglets. The birth weight and liver weight indicated disparate embryo growth and organ development in the two pig breeds, as the consequence of cell proliferation and differentiation. The present results agree with our previous findings that the serum cortisol in EHL was higher than that in lean type pigs [19]. The elevated serum TCH, HDLC, LDLC and higher liver content of TCH in EHL piglets indicated higher rate of cholesterol metabolism in this breed, which was in agreement with the enrichment of lipid metabolism related genes (Figure 4A). KEGG pathway analysis (Table S3) showed that there were 8 DEGs involved in peroxisome proliferator-activated receptor (PPAR) signal pathway. Among them, the higher expression of thrombospondin receptor (CD36) and fatty acid transporter (solute carrier family 27 member 2, SLC27A2) in EHL liver indicated increased response to serum LDLC and transport of HDLC. The lower level of CYP7A1 implicated lower synthesis of bile acid from cholesterol in the liver of EHL piglets, which may contribute to higher cholesterol deposition in liver [29]. By comparing the transcriptome of EHL and LW liver, we showed that a full scope of the liver metabolism processes varied between the two breeds of pigs.

The microarray used in our experiment could detect the miRNAs with RPM >10. For example, miR-193a-5p, which has an average RPM of 17, was detected readily by microarray. Nevertheless, the porcine miRNAs detected with deep sequencing often differ in sequence from their reference sequences in miRbase, as it was reported previously [6]. This may explain why some DEMs determined by sequencing could not be detected by microarray. The microarray probes were designed according to the miRbase sequences, whereas those abundantly expressed miRNAs (with an average RPM >10) that were undetectable by microarray had at least one nucleotide substitution compared to the miRbase sequences. Occasionally, some miRNAs with mismatches could also be detected by microarray. For example, miR-100, which has an average RPM of 958, was detected, however with the hybridization signal much lower than the perfectly matching miRNAs of the similar abundance. Besides, low copy miRNAs might also be undetectable by microarray due to low hybridization signals. Therefore, we combined the information obtained from deep sequencing and microarray formats to maximize the list of DEMs between EHL and LW piglets.

The coherent relationship between miRNA and mRNA is still under debate. Initial study showed that some miRNAs could induce reversed expression of mRNA and protein [30]. Actually, some recent studies showed that the uncoupling between mRNA and protein may implicate the post-transcription regulation mechanism [31]. Till now, there are 110 examples of mRNA cleavage and 178 examples of mRNA translation repression in Tarbase 5.0 (experimentally proved miRNA targets database) [32], [33]. But all the high-throughput experimental methods for identifying miRNA targets usually identify mRNAs or proteins which are down-regulated when a miRNA is over-expressed or vice versa [34]. It was indicated that the miRNA-induced destabilization of target mRNAs is the predominant reason for reduced protein output [35]. In agreement with the previous studies, we found that only the target genes with decreased expression corresponding to the up-regulated miRNAs were significantly enriched. Although 4 up-regulated miRNAs were found to be associated with the up-regulated targets (p<0.05), the correlation of up-regulated miRNAs and up-regulated mRNA was not statistically significant in general. So we integrated the reversely expressed targets of DEMs to delineate the regulatory mechanisms of miRNAs between EHL and LW piglets.

Most miRNAs in mammals pair imperfectly with their target mRNAs. Therefore it is difficult to seek their biologically important targets by the algorithm analysis alone [36]. Many algorithms are based on seed pairing–the paring of miRNA nucleotides 2–8. The miRanda, which was developed by miRbase, had the latest information of miRNA targets. Owing to the insufficiency of pig gene sequence annotation, the untranslated regions (UTRs) of many pig genes have not been identified. That is why we used the human genome information to generate our miRNA target list in the present study.

Compared to LW piglets, EHL piglets had more up-regulated miRNAs than down-regulated miRNAs in liver, as revealed by both deep sequencing (25 up-regulated, 15 down-regulated) and microarray (7 up-regulated, 3 down-regulated) methods. At the mRNA expression level, the figure went to opposite, i.e., there were less up-regulated than down-regulated mRNA genes (53 up-regulated, 200 down-regulated with FDR <5%, and 298 up-regulated, 510 down-regulated with FDR <10%). miRNAs are known to exert post-transcriptional regulation mostly by inducing mRNA degradation. Therefore, miRNAs are often expressed in an opposite pattern to the mRNA expression level of their target genes. Such inverse correlations between the expression of miRNAs and their target mRNAs are also observed in other studies [37], [38].

In an earlier study, the miRNA expression in liver was compared between Tongcheng (another Chinese indigenous breed) and Large White pigs at about 25 kg body weight by microarray [6]. Forty five miRNAs were found to be up-regulated, while only 13 were down-regulated in Tongcheng pigs. Among these DEMs, miR-133a, miR-451 and miR-739 are also identified as DEMs in the present study. The predominant up-regulated miRNA expression in the liver of Chinese indigenous pig breeds represented that the genesis of miRNA in these breeds was more active than that in LW. In fact, the enzymes involved in microRNA processing, Drosha and Dicer, were significantly up-regulated in EHL piglets than that in LW at protein level (data not shown).

The miRNA mediated processes are identified by analyzing the function of the target DEGs which were reversed correlated with DEM. Majority of the genes involved in DNA replication and protein translation were predicted to be the non-target genes of miRNA regulation (Figure 5), possibly because that genes participating in the DNA duplicating process or consisting ribosomes (ribosome proteins and histones) usually have short 3′UTR [39]. In the present study, the role of miRNAs in mediating the process of lipid metabolism was not evident, despite the fact that the differences in lipid metabolism are significant between the two pig breed. Nevertheless, miR-335, which was previously shown to be expressed more abundantly in the liver of obese mice [22], demonstrated higher hepatic expression in EHL piglets. In contrast, proteolysis is predicted to be a miRNA-mediated process. There are 28 DEMs targeting all the 14 genes involved in proteolysis, indicating that the process of proteolysis is regulated by the cooperation of many miRNAs. Among the 14 DEGs, 11 were down-regulated while 3 were up-regulated in EHL piglets. The decreased expression of most proteolysis related genes (11 of 14) in the liver of EHL indicated a weaker ability of protein turnover, and probably lower growth rate. However, mRNA expression is not directly related to the function. In some cases, the levels of mRNA and protein are reversely correlated. Therefore, it is possible that some proteolysis related DEGs are up-regulated (3 of 14) while the majority of the relevant genes are down-regulated. Nevertheless, direct evidences regarding the breed differences in hepatic proteolysis are needed to support our presumption.

In conclusion, we demonstrated the differences in the hepatic transcriptome and miRNAome between EHL and LW piglets with distinct phenotype and metabolic character. The most highly miRNA-mediated biological process with significant breed disparity is proteolysis. Our findings provide new information on the miRNA and mRNA profiles in porcine liver, and new approach in characterizing diverse traits in different pig breeds, thus serving as a basis for further investigation of the biological functions of miRNAs in porcine liver.

## Materials and Methods

### Animal Sampling

The newborn piglets were obtained from two neighboring pig breeding farms and sacrificed immediately after birth by exsanguination. The experiment was conducted following the guidelines of Animal Ethics Committee at Nanjing Agricultural University, China. The slaughter and sampling procedures complied with the “Guidelines on Ethical Treatment of Experimental Animals" (2006) No. 398 set by the Ministry of Science and Technology, China and “the Regulation regarding the Management and Treatment of Experimental Animals" (2008) No.45 set by the Jiangsu Provincial People’s Government. Six newborn male piglets from three litters (2 from each litter) of each purebred EHL and LW sows were sacrificed. Body weight, body length and liver weight were recorded. The blood was collected from the precaval vein and the serum was gathered and kept at −20°C. Liver samples were immersed in liquid nitrogen immediately after collection and then stored at −70°C.

### Measurement of Serum Biochemical Parameters

The measurement of serum glucose, TG, TCH, HDLC and LDLC was carried out by a service provider, Nanjing Jiancheng Bioengineering Institute.

Serum concentrations of leptin, insulin, cortisol, free T3 (FT3), total T3 and total T4 were measured by radioimmunoassay (RIA), using commercial kits (North Institute of Biotechnology, Beijing, China, Catalog: C16PDA, F01PZA, D10PZA, A03PZA, A01PZA, and A02PZA, respectively). The intra- and inter-assay coefficients of variation were below 5% and 10%, respectively, for all the six hormones.

All data were expressed as mean ± SEM. The p-value was calculated by the student t-test for independent samples with the SPSS 13.0 for Windows. False discovery rates (FDRs) were calculated using the method of Benjamini & Hochberg (1995) [40]. FDR <0.05 was considered significant.

### RNA Isolation

Total RNA was isolated from liver,using the Trizol reagent (Invitrogen, USA), according to the manufacturer’s instructions. Concentration of the extracted RNA was measured using NanoDrop ND-1000 Spectrophotometer [41]. RNA integrity was confirmed by denaturing agarose electrophoresis, and DNA contamination was evaluated by PCR using isolated RNA as template with the primers of GAPDH (primer sequences are shown in Table S4). High quality RNA samples were then stored at −70°C till further use.

### Small RNA Library Preparation and Sequencing

The total RNA samples were prepared as follows: 2000 ng of total RNAs from the twelve pigs were isolated and pooled within each breed (EHL and LW). Both small RNA libraries were generated according to Illumina’s sample preparation instruction. Then they were sequenced on the Illumina GAIIx (Illumina, USA) following vendor’s instruction. Raw sequencing reads were obtained using Illumina’s Pipeline v1.5 software following sequencing image analysis by Pipeline Firecrest Module and base-calling by Pipeline Bustard Module.

The reads were then subjected to a series of data filtration steps to obtain mappable sequences using ACGT101-miR v3.5 (LC Sciences, USA) with the statistics of mammalian miRNAs in miRBbase 16.0.

### miRNA Microarray

Total RNAs of the two littermate piglets were pooled for the microarray analysis. The pooled samples were named as EHL1–3 (n = 3) and LW1-3 (n = 3).

The pig microRNA microarray was obtained from LC Sciences (Houston, USA) and contains 238 unique probes that were complementary to all mature miRNAs of pig in miRBase release 16.0. The assay started from 5 µg total RNA sample, which was size fractionated using a YM-100 Microcon centrifugal filter (Millipore, USA) and the small RNAs (<300 nt) isolated were 3′-extended with a poly(A) tail using poly(A) polymerase. An oligonucleotide tag was then ligated to the poly(A) tail for later fluorescent dye staining. Hybridization was performed overnight on a µParaflo microfluidic chip using a micro-circulation pump (Atactic Technologies, USA). On the microfluidic chip, each detection probe consisted of a chemically modified nucleotide coding segment complementary to target miRNAs (from miRBase, release 16, 238 probe sets) and a spacer segment of polyethylene glycol to extend the coding segment away from the substrate. The detection probes were made by in situ synthesis using photogenerated reagent (PGR) chemistry. The hybridization melting temperatures were balanced by chemical modifications of the detection probes. 100 µL 6×SSPE buffer containing 25% formamide was used for hybridization at 34°C. After hybridization, fluorescence labeling using tag-specific Cy5 dyes was used for detection. Hybridization images were collected using a laser scanner (GenePix 4000B, Molecular Device) and digitized using Array-Pro image analysis software (Media Cybernetics). Data were analyzed by first subtracting the background and then normalizing the signals using a LOWESS (locally weighted regression) function. The three samples of LW piglets were used as control group to analysis the different expression between the two pig lines. The differences between the two groups were analyzed using SAM (Significance Analysis of Microarrays) method with SAMR software version 3.02 [42]. The FDR (False Discovery Rate) and fold change were calculated. miRNAs with FDR <15% were considered to be differentially expressed miRNAs. All the microarray data were MIAME compliant and have been deposited in GEO (accession number GSE33523).

### Real-time qPCR Confirmation of Different Expressed miRNAs

Two µg of total RNA from each piglet were polyadenylated by poly(A) polymerase (PAP) at 37°C for 1 h in a 20 µL reaction mixture using Poly(A) Tailing Kit (AM1350, Ambion, USA) according to the manufacturer’s instructions. Treated RNA was then dissolved and reverse-transcribed using poly (T) adapter and M-MLV (Promega, USA). qPCR for the 18 miRNAs was performed using SYBR Green Real-time PCR Master Mix (TaKaRa, Japan) in Mx3000P (Stratagene, USA) with a miRNA specific forward primer and a universal reverse primer complementary to part of the poly(T) adapter sequence. U6 was chosen as an internal control to normalize the technical variations. The sequences for all the primers and the poly (T) adapter are listed in Table S4. The method of 2^−ΔΔ^CT was used to analyze the real-time PCR data expressed as the fold change relative to the average value of the LW piglets [43]. The differences between the two groups were analyzed by the student t-test for independent samples with the SPSS 13.0 for Windows.

### mRNA Microarray Experiment

The samples used for mRNA microarray were the same as the miRNA microarray. Microarray was performed by a service provider (CapitalBio, China). The microarray (Probe length 60-mer, 135K Format) containing 44987 probe sets was provided by Roche-NimbleGen. For each sample, 1 µg of total RNA was treated with the CapitalBio cRNA Amplification and Labeling Kit (CapitalBio, China) according to the manufacturer’s instructions. After reverse transcribed with a T7 oligo(dT) primer, second-strand synthesis and purification, the generated cDNAs were in vitro transcribed to synthesize multiple copies of cRNAs. Then 5 µg of purified cRNAs were reverse transcribed with random primer. Labeled cDNA molecules were generated by subsequent Klenow Fragment Polymerase labeling with Cy3-dCTP (GE Healthcare, USA). Following purification and drying, the labeled cDNAs were then dissolved in 80 µl hybridization buffer (3×SSC, 0.2%SDS, 5×Denhart’s, 25% formamide). Hybridizations were performed overnight at 42°C using hybridization system 12 (Roche NimbleGen, USA). The arrays were then washed and dried. The fluorescence intensity was collected using NimbleGen MS 200 Microarray Scanner. Data were extracted from scanned images using NimbleScan v2.6 software. Quantile normalization RMA (Robust Multi-Array) analysis was performed to generate gene expression values. The differences between the two groups were analyzed using SAM (Significance Analysis of Microarrays) method with SAMR software version 3.02 [42]. The FDR (False Discovery Rate) were calculated. Differentially expressed genes (DEGs) were selected with FDR <5% and FDR <10%. All data were MIAME compliant and have been deposited in GEO (accession number GSE33524).

### Bioinformatics and Statistical Analysis

The target genes of miRNAs were predicted by miRanda algorithm. Correlation analysis of the miRNA and mRNA expression profiles was carried out using the lists of DEMs and DEGs. The significance of all the 4 types of possible miRNA-mRNA correlations (up-regulated miRNA and up-regulated mRNA, up-regulated miRNA and down-regulated mRNA, down-regulated miRNA and down-regulated mRNA, down-regulated miRNA and down-regulated mRNA) were analyzed using two tailed chi-square test.

A two-tailed Fisher Exact Test was conducted for each DEM to determine whether the number of predicted target genes that were differentially regulated was higher than would be expected by chance. The Fisher Exact test was conducted for each miRNA using both down-regulated and up-regulated DEG lists.

The GO and KEGG pathway analysis were carried out by using a Molecule Annotation System called MAS 3.0 (http://bioinfo.capitalbio.com/mas3/) and the enrichment p-values were calculated.

The DEG list with FDR <10% was used to characterize the function of miRNA-mediated DEGs. We classified the DEGs into two categories, coherent targets and non-coherent targets. Coherent target genes are predicted DEM target genes that are negatively correlated with the expression of DEMs. For each GO term, the percentages of the coherent and non-coherent targets were compared using a two-tailed chi-square test.

## Supporting Information

Table S1 **All conserved miRNAs detected by sequencing with RPM >10.**
(XLS)

Table S2 **List of all DEGs selected at FDR<10%.**
(XLS)

Table S3 **KEGG pathway analysis of DEGs selected at FDR<10%.**
(XLS)

Table S4 **List of primers used for miRNA detection.**
(DOC)
