# Supplementary material for: Coordinated miRNA/mRNA Expression Profiles for Understanding Breed-Specific Metabolic Characters of Liver between Erhualian and Large White Pigs
Source: PLoS One. 2012 Jun 12;7(6):e38716. doi: 10.1371/journal.pone.0038716 (PMC3373568; doi:10.1371/journal.pone.0038716)
Supplement: Table S4 — List of primers used for miRNA detection. (DOC) [file pone.0038716.s004.doc]

**Table S4 primers used for miRNA detection**

| Name | Primer sequence (5’to 3’) | miRbase Acc No. |
| --- | --- | --- |
| miR-130b | CAGTGCAATGATGAAAGGGCAT | MIMAT0013922 |
| miR-15b | TAGCAGCACATCATGGTTTACA | MIMAT0002125 |
| miR-184 | TGGACGGAGAACTGATAAGGGT | MIMAT0002127 |
| miR-185 | TGGAGAGAAAGGCAGTTCCTGA | MIMAT0007759 |
| miR-193a-5p | TGGGTCTTTGCGGGCGAGATGA | MIMAT0013894 |
| miR-221 | AGCTACATTGTCTGCTGGGTTT | MIMAT0007762 |
| miR-27a | TTCACAGTGGCTAAGTTCCGC | MIMAT0002148 |
| miR-378 | ACTGGACTTGGAGTCAGAAGGC | MIMAT0013868 |
| miR-500 | ATGCACCTGGGCAAGGATTCT | MIMAT0013956 |
| miR-532-5p | CATGCCTTGAGTGTAGGACCGT | MIMAT0013940 |
| miR-222 | AGCTACATCTGGCTACTGGGTCT | MIMAT0013942 |
| miR-574 | CACGCTCATGCACACACCCACA | MIMAT0013951 |
| miR-100 | CAAGCTTGTGTCTATAGGTAT | MIMAT0013911 |
| miR-216 | TAATCTCAGCTGGCAACTGTG | MIMAT0002130 |
| miR-146a | TGAGAACTGAATTCCATGGGTT | MIMAT0017971 |
| miR-652 | AATGGCGCCACTAGGGTTGTG | MIMAT0017964 |
| miR-133a | TTGGTCCCCTTCAACCAGCTG | Seq result |
| miR-582-5p | TTACAGTTGTTCAACCAGTTACT | Seq result |
| oligodT adaptor | TAGAGTGAGTGTAGCGAGCACAGAA  TTAATACGACTCACTATAGGTTTTTT  TTTTTTTTTTVN | N/A |
| universal primer | TAGAGTGAGTGTAGCGAGCA | N/A |
| U6 | GGCAAGGATGACACGCAAAT | ENSSSCT00000019750 |
